# The Combination of Start-Codon-Targeted (SCoT) and *Falling Stone* (*FaSt*) Transposon-Specific Primers Provides an Efficient Marker Strategy for *Prunus* Species

**DOI:** 10.3390/ijms26093972

**Published:** 2025-04-23

**Authors:** Beti Ivanovska, Thanyarat Onlamun, Júlia Halász, Attila Hegedűs

**Affiliations:** Group of Horticultural Plant Genetics, Department of Plant Biotechnology, Institute of Genetics and Biotechnology, Hungarian University of Agriculture and Life Sciences, Ménesi út 44., 1118 Budapest, Hungary

**Keywords:** genetic variability, genome evolution, MITE, molecular markers, phylogenetic analysis, stone fruits, transposon

## Abstract

A novel primer (FaSt-R) targeting the *Prunus*-specific *Falling Stone* (*FaSt*) non-autonomous transposon was combined with start-codon-targeted (SCoT) primers to assess genetic diversity in 12 cultivars from six *Prunus* species and 28 cultivars of European plum. Compared to SCoT-only analyses, the SCoT–FaSt combination produced fewer total bands but a higher percentage of polymorphic bands, while maintaining comparable values for polymorphism information content, resolving power, gene diversity, and Shannon’s index. SCoT–FaSt markers generated bands across a broader size range, which made gel patterns less dense, enabling the more accurate detection of differentially amplified fragments. Neighbor-joining and principal component analyses confirmed that SCoT–FaSt markers provided sufficient phylogenetic resolution at both interspecific and intraspecific levels. The sequencing of 32 SCoT–FaSt amplicons revealed *FaSt* elements in 26 fragments, with SCoT primers preferentially annealing to GC-rich exonic and intergenic regions. Seventeen protein-coding and one RNA-coding gene were partially identified, with *FaSt* elements localized in UTRs and introns of genes with key physiological functions. Comparative analysis indicated a biased distribution of *FaSt* elements between the *Cerasus* and *Prunus* subgenera. In silico findings suggest that *FaSt* elements are more fragmented in cherry species, potentially contributing to subgeneric divergence. Overall, the SCoT–FaSt marker system is effective for evaluating *Prunus* genetic diversity, reconstructing phylogenetic relationships, and elucidating the genomic impact of an active Mutator-like transposon.

## 1. Introduction

The genus *Prunus* (Rosaceae, subfamily Amygdaloideae) comprises many economically significant temperate fruit trees, including peach [*P. persica* L. (Batsch)], almond (*P. dulcis* Mill.), apricot (*P. armeniaca* L.), sweet cherry (*P. avium* L.), sour cherry (*P. cerasus* L.), and European plum (*P. domestica* L.) [1]. These species provide valuable fresh and processed fruits rich in sugars, minerals, antioxidants, and other bioactive compounds [2]. Traditionally divided into five subgenera based on morphology [3], *Prunus* is now commonly grouped into three subgenera—*Prunus*, *Cerasus*, and *Padus*—according to molecular evidence [4]. Phylogenomic studies using RAD-seq and genome skimming have clarified the evolutionary relationships within the genus, particularly inflorescence diversification [5]. Inflorescence type correlates with subgenus: *Prunus* species have solitary flowers, *Cerasus* have corymbose, and *Padus* display racemose structures [6]. Most species in *Prunus* and *Cerasus* are diploid (e.g., almond, peach, apricot, sweet cherry), although polyploid species also exist, such as tetraploid sour cherry and hexaploid European plum.

The *Prunus* genus likely originated in eastern Asia around 61 million years ago (Mya) and diversified through climatic and geological shifts that shaped its current distribution [6]. The *Cerasus* and *Prunus* lineages diverged approximately 54 Mya. Sour cherry (*P. cerasus*) is an allotetraploid hybrid of *P. fruticosa* and *P. avium* [7,8], while apricot and plums, including European plum (*P. domestica*), diverged from a common ancestor about 35–40 Mya [6]. *P. domestica* likely arose from hybridization between *P. cerasifera* and *P. spinosa*, though its hexaploid genome complicates precise parentage resolution [9]. Peach and almond, which diverged 8–5.8 Mya, share a common ancestor and differentiated under contrasting water availability conditions in Central Asia [10,11,12].

Various molecular marker systems have been used to assess genetic diversity in fruit trees. PCR-based techniques, such as amplified fragment length polymorphism (AFLP), random amplified polymorphic DNA (RAPD), inter-simple sequence repeat (ISSR), and especially microsatellite (SSR) markers, have largely replaced hybridization-based methods. SSRs have been extensively applied in *Prunus* genetic studies [13,14,15,16,17,18] due to their codominant nature. However, as they are primarily located in non-coding genomic regions, their association with phenotypic traits is often limited [19].

Start-codon-targeted (SCoT) markers have recently emerged as a promising tool for analyzing genetic relationships, particularly due to their simplicity, high polymorphism, and ability to target coding regions using single primers [20]. Despite their advantages, SCoT markers remain underutilized in *Prunus* research. Limited studies have demonstrated their potential: SCoT analysis revealed high genetic diversity in *P. sibirica* populations in Inner Mongolia [21], while Thakur et al. [22] found SCoT markers to have the highest polymorphic information content compared to RAPD and ISSR in Japanese plum. Similarly, Atapour et al. [23] reported strong discriminatory power in *P. avium* genotypes using 12 SCoT markers.

Transposable elements (TEs) are broadly classified into Class I (retrotransposons) and Class II (DNA transposons) [24]. Class I elements propagate via an RNA intermediate using a “copy-and-paste” mechanism, often accumulating in high copy numbers within repetitive genomic regions. In contrast, the less abundant Class II elements move via a “cut-and-paste” process, relying on a transposase enzyme, terminal inverted repeats (TIRs), and target site duplications (TSDs) [25]. Miniature inverted-repeat transposable elements (MITEs) are non-autonomous Class II elements characterized by small size (≤600 bp), conserved TIRs, A/T-rich sequences, and specific TSDs [24]. Despite lacking coding potential, MITEs often reach copy numbers exceeding those of their autonomous counterparts [26].

Advances in high-throughput sequencing have enabled detailed analysis of repetitive elements in *Prunus* genomes. In *P. persica*, transposable elements (TEs) comprise 29.6% of the genome, including 18.6% long terminal repeat (LTR) retrotransposons and 9.1% DNA transposons [27]. Similarly, *P. mume* contains 27.8% LTRs and 8.5% DNA transposons [28], while *P. avium* has 6.4% LTRs and 2.6% DNA elements [29]. In *P. dulcis* cv. ‘Texas’ (v3.0), TEs account for 33.0% of the genome, with 24.3% retrotransposons and 8.7% DNA transposons [26].

Due to their abundance in plant genomes, retrotransposons have been the primary targets for TE-based molecular markers, including inter-retrotransposon amplified polymorphism (IRAP) and inter-primer binding site (iPBS) amplification [30,31]. These markers detect LTR insertion polymorphisms and are typically dominant. DNA transposons, by contrast, are less frequently targeted. Their detection has been enabled by transposon display (TD), a modified AFLP approach, with miniature inverted-repeat transposable elements (MITEs) commonly used in MITE-TD. However, TE-based marker applications in *Prunus* remain limited and have thus far focused exclusively on retrotransposon-derived systems [32,33,34].

A MITE named *Falling Stones* (*FaSt*) was identified in *Prunus armeniaca* [35]. With a length of 349 bp, 82 bp TIRs, and 9 bp TSDs, *FaSt* is likely a member of the Mutator transposon superfamily. It preferentially accumulates in AT-rich, gene-dense regions across all peach chromosomes. Functionally, *FaSt* insertions have been implicated in the breakdown of self-incompatibility in apricot. Specifically, a nested insertion into the *S-haplotype-specific F-box* gene led to the breakdown of self-incompatibility, while another insertion at the *M-locus disulfide bond A-like* gene in self-compatible cultivars ‘Canino’ and ‘Katy’ resulted in the disruption of the open reading frame and consequent self-compatibility [36].

While SCoT markers have gained popularity for detecting sequence variation in coding regions, their single-primer design poses certain limitations. Given that the *FaSt* transposon preferentially inserts within or near genes in the *Prunus* genome, we investigated whether combining SCoT and *FaSt*-specific primers could enhance marker efficiency. This study compared the polymorphism levels and amplicon sizes generated by SCoT or *FaSt* primers, and their combinations across multiple *Prunus* species and a diverse set of *P. domestica* cultivars. Our objectives were to assess the utility of SCoT–FaSt marker combinations for evaluating genetic diversity, resolving phylogenetic relationships, and to determine the genomic regions targeted by these markers through the cloning and sequencing of selected amplicons.

## 2. Results

### 2.1. Rationale and Basic Concept

**Figure 1:**
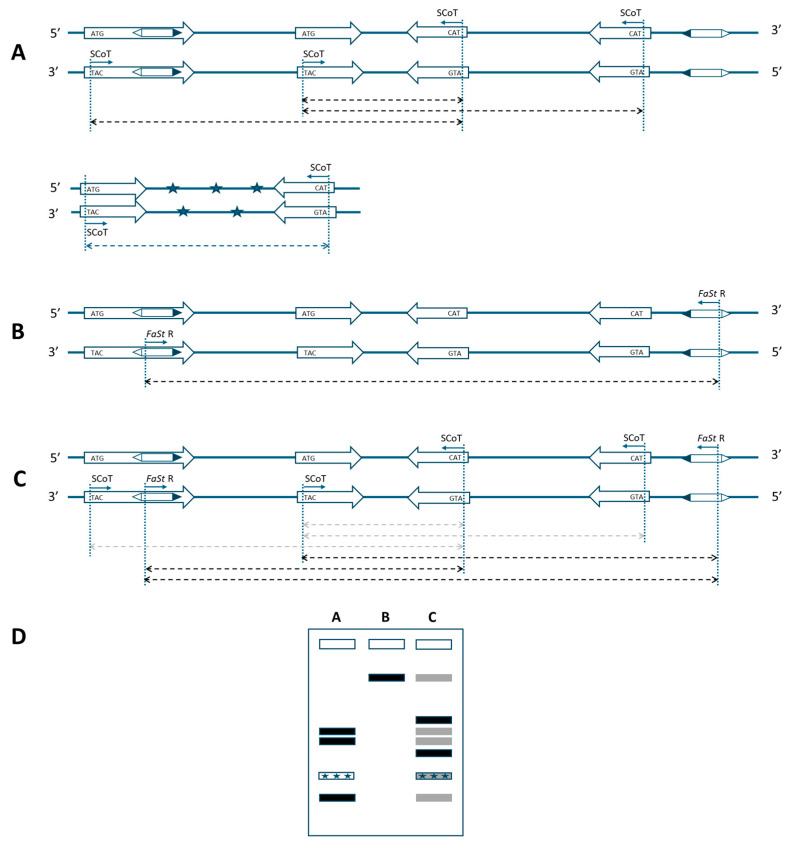
Schematic representation of a marker system based on start-codon-targeted (SCoT) and *Falling Stones* (*FaSt*)-specific primers for *Prunus* species. (**A**) Amplification in the original SCoT assay requires two genes in opposite orientation within an amplifiable distance. (**B**) A single FaSt-R primer can also yield amplicons under similar conditions, due to the high copy number and varied orientation of *FaSt* elements. (**C**) The combination of SCoT and FaSt-R primers can amplify fragments if a *FaSt* element is located in the proper orientation near a gene. As FaSt-R is sequence-specific, SCoT–FaSt combinations are expected to outperform the consensus SCoT-only assays and also amplify more reliably than primers annealing to the distantly located *FaSt* elements (weak amplification indicated by gray arrows). Thick arrows: genes in 5′ to 3′ orientation; hexagons: *FaSt* elements with black triangles marking the 5′ end; thin arrows: primer annealing sites; dashed arrows: expected amplicons; asterisks: non-target DNA. (**D**) Representative banding patterns from the described PCR strategies. Gray bands indicate weak or undetectable amplification.

**Figure 2:**
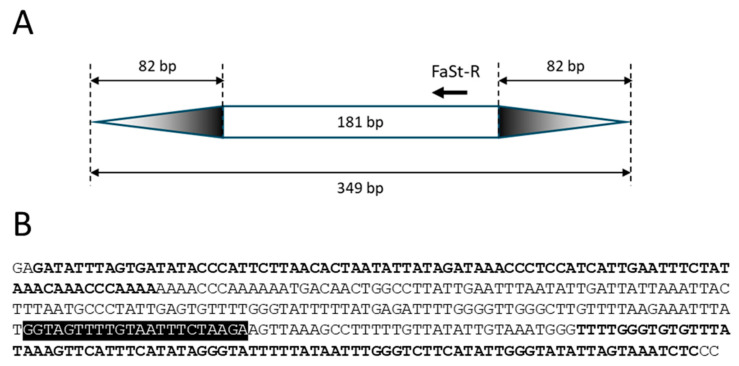
Structure and sequence of the *Prunus armeniaca Falling Stone* (*FaSt*) transposon (KF956794). (**A**) Schematic representation of the non-autonomous *FaSt* element, with structural motifs shown to scale. Terminal inverted repeats (TIRs) are indicated by gradient-colored triangles, and the annealing site and 5′→3′ orientation of the FaSt-R primer are marked with an arrow. (**B**) Nucleotide sequence of the *FaSt* element. TIRs are highlighted in bold, and the FaSt-R primer binding site is shown against a black background.

Single SCoT primers only enable amplification when two genes are positioned within a suitable distance and in opposite orientation (Figure 1A). This constraint also applies to the FaSt-R primer developed in this study. The 349 bp *FaSt* element comprises two terminal inverted repeats (TIRs) of 82 bp each, which were intentionally avoided during primer design to prevent the amplification of a single dominant band from the numerous *FaSt* copies in the *Prunus* genome. Instead, the FaSt-R primer was designed to target a 22-nucleotide region between positions 212 and 233 within the internal sequence of the *FaSt* element (Figure 2). Owing to the high copy number and variable orientation of *FaSt* insertions across the *Prunus* genome, the FaSt-R primer can also function effectively in a single-primer PCR assay (Figure 1B).

When combined, SCoT and FaSt-R primers are expected to preferentially amplify genomic regions located between genes and nearby *FaSt* elements, as the FaSt-R primer specifically anneals to unique internal regions of *FaSt* copies (Figure 1C). Although amplification with individual SCoT or FaSt primers may remain possible, fragments amplified more efficiently by the combined approach may predominate, while weaker products become depleted. The sequence specificity of the FaSt-R primer also reduces the likelihood of amplifying contaminating DNA, as *FaSt* elements are exclusively found in *Prunus* (Figure 1A). Given the preferential localization of *FaSt* elements in introns and untranslated regions (UTRs), the combined strategy may also result in shorter amplicon sizes.

### 2.2. Amplification Efficiency of SCoT, FaSt-R, and Combined Primer Sets

**Figure 3:**
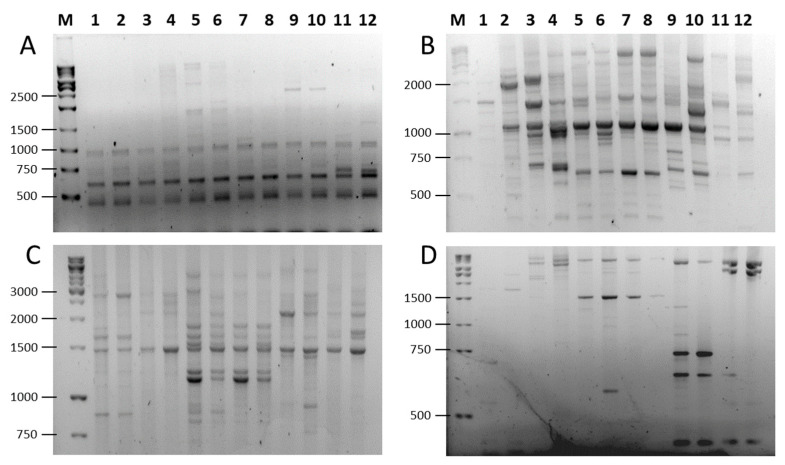
PCR amplification patterns in six *Prunus* species using SCoT and combined SCoT (forward) and FaSt-R (reverse) primers. (**A**) SCoT23 alone; (**B**) SCoT23 and FaSt-R; (**C**) Scot27 alone; (**D**) SCoT27 and FaSt-R. Lanes are labeled as follows: (M) GeneRuler 1 kb DNA ladder, *P. armeniaca* (1) ‘Ceglédi óriás’ and (2) ‘Gönci magyar kajszi’; *P. dulcis* (3) ‘Tuono’ and (4) ‘Tétényi keményhéjú’; *P. cerasus* (5) ‘Kántorjánosi 3′ and (6) ‘Feketicsi meggy’; *P. avium* (7) ‘Canada giant’ and (8) ‘Katalin’; *P. persica* (9) ‘Collins’ and (10) ‘Condor’ *P. domestica* (11) ‘Toptaste’ and (12) ‘Haroma’.

To assess the amplification efficiency of SCoT, FaSt-R, and their combinations, we analyzed two cultivars from each of six *Prunus* species (*P. persica*, *P. dulcis*, *P. armeniaca*, *P. avium*, *P. cerasus*, and *P. domestica*). We used 19 SCoT primers in single-primer assays. The FaSt-R primer was also tested independently and in combination with each SCoT primer. In many cases, the inclusion of FaSt-R improved the clarity and quality of banding patterns (Figure 3). For instance, while SCoT 23 alone produced largely monomorphic fragments (Figure 3A), the addition of FaSt-R increased the percentage of polymorphic bands (Figure 3C). Similarly, SCoT 27 generated numerous weak and diffuse bands (Figure 3B), but its combination with FaSt-R yielded fewer, yet more intense and clearly resolved bands, which were easier to interpret (Figure 3D).

The use of the FaSt-R primer alone yielded the lowest values across all evaluated parameters, including total number of scorable bands (TNB), number of distinct band sizes (NDB), percentage of polymorphic bands (PPB), resolving power (*Rp*), Nei’s gene diversity (*h*), and Shannon’s information index (*I*) (Table 1). Additionally, the smallest fragment amplified by FaSt-R alone was 700 bp, substantially larger than the minimum fragment sizes observed for SCoT alone (250 bp) and SCoT–FaSt combinations (150 bp). SCoT-only assays produced the highest average TNB, percentage of amplicons ≤1000 bp, and *Rp* values. In contrast, while the SCoT–FaSt combinations resulted in slightly lower TNB values, they exhibited considerably higher PPB. The values of NDB, polymorphism information content (PIC), *h*, and *I* were comparable between SCoT and SCoT–FaSt assays.

TNB was higher for 14 of the 19 SCoT primers compared to the corresponding SCoT–FaSt combinations, improving gel evaluation. In cases with fewer bands, band intensity and sharpness were improved, as observed for SCoT 27-FaSt-R (Figure 3D). Although the average percentage of amplicons ≤1000 bp was higher for SCoT primers than for the SCoT–FaSt combinations, two primers (SCoT 7 and SCoT 11) produced small amplicons only when combined with FaSt-R. Additionally, six primers (SCoT 2, 13, 19, 21, 22, and 34) showed a significant increase in the number of small amplicons when paired with FaSt-R. The PPB remained at 100% for two SCoT primers and increased for all but two primers (SCoT 13 and 24) when combined with FaSt-R. The inclusion of FaSt-R decreased the PIC for 12 SCoT primers, with reductions ranging from 3% to 25%, while the remaining primers exhibited a slight increase in PIC. The *Rp*, *h* and *I* increased for 7 of the 19 SCoT primers when combined with FaSt-R. Notably, the addition of FaSt-R led to substantial improvements in some cases, such as a 339% increase in *Rp* for SCoT 11 and a 50% increase in *h* for SCoT 2.

### 2.3. Phylogenetic Analysis of Prunus Using SCoT–FaSt Markers

**Figure 4:**
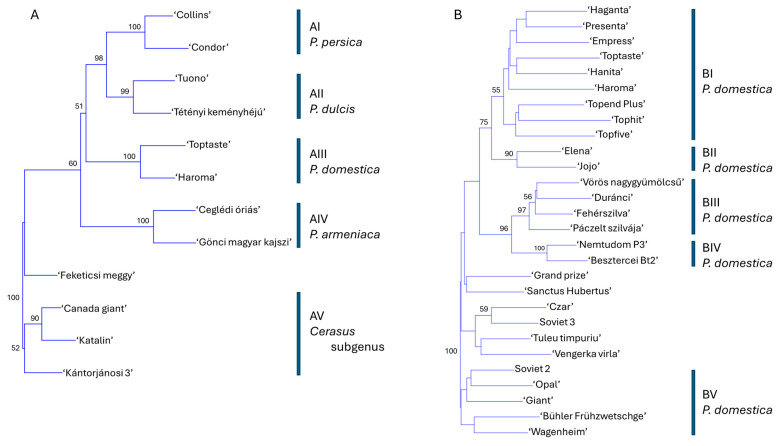
Phylogenetic analysis of *Prunus* based on data from 19 SCoT–FaSt primer combinations using the neighbor-joining method and Jaccard’s similarity coefficients. (**A**) Twelve cultivars representing six *Prunus* species: *P. dulcis*, *P. armeniaca*, *P. domestica*, *P. persica*, and members of the *Cerasus* subgenus (*P. avium* and *P. cerasus*). (**B**) Twenty-eight *P. domestica* cultivars. Bootstrap values (≥50%) from 1000 replicates are shown at the corresponding nodes. Groups AI–AV and BI–BV denote statistically supported clusters of cultivars.

A phylogenetic tree was constructed using data from 19 SCoT–FaSt marker combinations to evaluate the genetic relationships among 12 cultivars representing six *Prunus* species. The analysis yielded five major clusters, each supported by 95–100% bootstrap values, corresponding to *P. persica* (AI), *P. dulcis* (AII), *P. domestica* (AIII), *P. armeniaca* (AIV), and species within the *Cerasus* subgenus (AV) (Figure 4A). Within the *Cerasus* cluster, *P. avium* cultivars formed a well-supported subgroup, while ‘Feketicsi meggy’ was positioned separately. Notably, the *P. persica* and *P. dulcis* clusters formed a joint group with high bootstrap support, indicating closer genetic relatedness to each other than to *P. domestica*, *P. armeniaca*, or the *Cerasus* species. Higher ploidy levels in *P. cerasus* and *P. domestica* did not correspond with an increased number of detected alleles compared to diploid species. A similar phylogenetic analysis was performed using data from the SCoT-only assay (Figure S1). While the overall tree structure was comparable, a key difference emerged: *P. persica* and *P. dulcis* did not cluster together. Instead, *P. persica* appeared more closely related to *P. domestica* in this analysis.

Given the robustness of the evolutionary inferences derived from the SCoT–FaSt amplification patterns, we extended the analysis to include SCoT–FaSt amplicon data from 28 *P. domestica* cultivars (Figure 4B; Figure S2). The resulting phylogenetic tree revealed five major clusters. Cluster BI comprised modern German cultivars along with several of their progenitors used in their breeding programs. Cluster BII contained two offspring of the cultivar ‘Stanley’. Clusters BIII and BIV shared a common origin and encompassed all Hungarian landraces. Cluster BV included older cultivars from Germany, Russia, Sweden, and the USA. An additional statistically unsupported cluster contained cultivars from the UK, Russia, and Romania.

**Figure 5:**
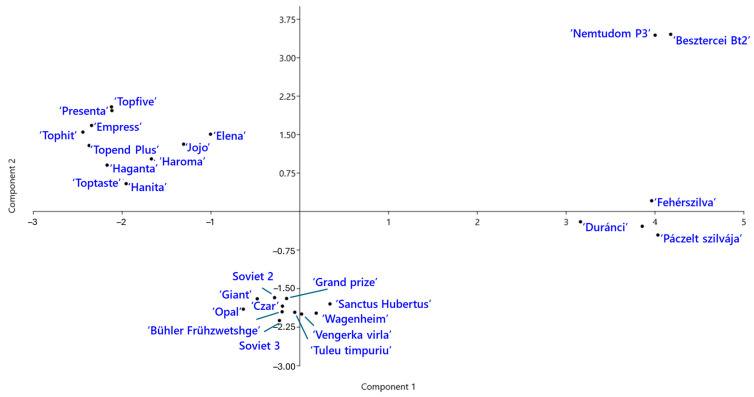
Principal component analysis (PCA) of *Prunus domestica* cultivars based on SCoT–FaSt marker data. The plot shows the distribution of cultivars of different origins along the first two principal components.

Genetic relationships among the *P. domestica* cultivars were further explored using principal component analysis (PCA) (Figure 5). The first two components explained 23.8% and 14.8% of total genetic variation, respectively. The PCA plot revealed four distinct groups of cultivars. ‘Jojo’ and ‘Elena’ clustered with the modern German cultivars, suggesting genetic similarity. Notably, the two Hungarian landraces, ‘Besztercei Bt2’ and ‘Nemtudom P3’, were located near each other but clearly separated from the remaining Hungarian landraces along the second principal component.

### 2.4. Sequence Analysis of the Amplified Fragments

To determine whether the amplified fragments contained recognizable *FaSt* elements and genic regions, a subset of amplicons was cloned and sequenced. A total of 32 sequences were submitted to the National Center for Biotechnology Information (NCBI) GenBank under accession numbers PQ869779–PQ869809. These sequences were generated using the FaSt-R primer in combination with the following SCoT primers: SCoT1 (2 sequences), SCoT2 (4), SCoT3 (2), SCoT4 (8), SCoT16 (1), SCoT19 (1), SCoT21 (1), SCoT22 (7), SCoT32 (3), and SCoT33 (3). The cloned fragments originated from the following *Prunus* species: *P. domestica* (11 fragments), *P. cerasus* (9), *P. dulcis* (8), *P. persica* (2), *P. avium* (1), and *P. armeniaca* (1).

Among the 32 sequenced fragments, 26 contained a recognizable *FaSt* element (58 to 218 bp). In six sequences, the FaSt-R primer annealed to a degenerate complementary region not associated with a *FaSt* element. In these cases, the primer binding sites were located within the 3′ untranslated region (UTR) in four sequences (*Pcer_KJ_2_1227*, *Pcer_FM_2_1227*, *Pcer_KJ_4_965*, and *Pdom_EL_4_766*), and within introns in two sequences (*Pcer_KJ_4_662* and *Pdom_TT_21_269*). These sequences were associated with genes encoding proteins such as β-D-glucosyl crocetin β-1,6-glucosyltransferase, peroxidase N1, methyltransferase PMT10, deoxyloganetin glucosyltransferase, and WUSCHEL-related homeobox 9.

Of the 26 identified *FaSt* elements, 11 were located within genic regions, specifically in the 5′ UTR (*Pdom_1-TF-907*, *Pdom_BB_1_908*, and *Pdul_TU_3_544*), 3′ UTR (*Pdom_NT_22_873* and *Pdom_HR_32_1224*), or introns (*Pdul_TK_4_761*, *Pdul_TK_22_901*, *Pcer_KJ_4_1000*, *Pcer_FM_4_496*, *Pdom_NT_22_334*, and *Pdom_HR_33_1460*). They exhibited significant homology (*E* values ranging from 0.0 to 7 × 10^−14^) to genes encoding polygalacturonase, inositol 2-dehydrogenase/D-chiro-inositol 3-dehydrogenase, BTB/POZ domain-containing protein, pectinesterase, DEAD-box ATP-dependent RNA helicase 52C, ethylene-responsive transcription factor, glycosyltransferase, β-D-glucosyl crocetin β-1,6-glucosyltransferase, leaf rust 10 disease resistance locus receptor-like protein kinase, and one uncharacterized protein (Table S1). One *FaSt* element (*Pcer_FM_4_1056*) was located near a *U1* spliceosomal RNA coding gene and the remaining 15 *FaSt* elements were in intergenic regions (Table S1).

**Figure 6:**
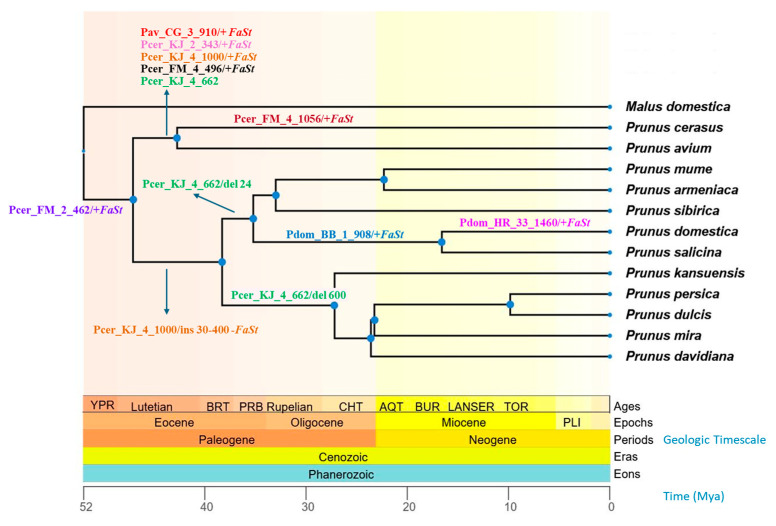
Chronogram of 12 *Prunus* species and one *Malus* species based on divergence time estimation from the TimeTree 5 database [37]. The geological timescale is indicated by background colors and shown in millions of years ago (Mya) below the cladogram. Arrows indicate *Falling Stones* (*FaSt*) transposon-induced sequence alterations (+ presence and − absence of *FaSt*; “ins” and “del” refer to other sequence insertions and deletions, respectively). Sequence identifiers are provided in Table S1.

Selected examples showed interesting patterns of conservation or divergence across *Prunus* species. For instance, two sequences, *Pdom_1-TF-907* and *Pdom_BB_1_908*, showed homology to the *Prunus persica polygalacturonase ADPG2* gene, while the embedded *FaSt* element aligned independently with several *P. persica FaSt* sequences. BLASTn analysis against the *P. domestica* genome revealed 98% identity with two gaps across a 936 bp alignment. The *FaSt* element was located within the 5′ UTR, upstream of the start codon of the open reading frame. This sequence, including the *FaSt* insertion, was conserved in both ‘Topfive’ and ‘Besztercei Bt.2’ cultivars, and was also present in the *P. salicina* genome. However, the *FaSt* element was absent from all other *Prunus* species examined (Figure 6).

The complete sequence of *Pav_CG_3_910* was found in both the sweet and sour cherry genomes, but its *FaSt* element was absent in *P. fruticosa*, *P. armeniaca*, *P. persica*, and all other *Prunus* species (Figure 6). Similarly, the *Pcer_KJ_2_343* sequence exhibited 48% coverage with its closest homolog in BLASTn searches. However, when blasted against the sour and sweet cherry genomes on the Genome Database of Rosaceae (GDR) platform, the full sequence was detected (*E* value 0.0, 100% identity). This sequence was absent from *P. fruticosa* and all other *Prunus* species analyzed.

The *Pcer_KJ_4_1000* sequence spans the intron of a gene encoding a BTB/POZ domain-containing protein and contains a *FaSt* element within this intronic region. This sequence was present in the *P. cerasus* and *P. avium* genomes, but in *P. fruticosa* and *P. yedonensis*, a small insertion (~60 bp) was observed within the intron, outside the *FaSt* element. In *P. campanula*, the insertion was somewhat larger (~200 bp). The genomes of *P. salicina*, *P. mongolica*, *P. zhengheensis*, *P. dulcis*, *P. persica*, *P. armeniaca*, and *P. × kanzakura* lacked the *FaSt* element but contained insertions ranging from 30 to 400 bp.

The *Pcer_FM_4_496* sequence, located within the intron of a *pectinesterase* gene, contained a *FaSt* element that was found only in *P. speciosa*, *P. avium*, and *P. cerasus*, while other more distantly related species (e.g., *P. dulcis* and *P. salicina*) did not possess this *FaSt* copy. The *FaSt* element in *Pcer_FM_4_1056* was absent from homologous sequences in *P. avium*, *P. persica*, and *P. armeniaca*. Notably, the ‘Montmorency’ snRNA gene sequence exhibited a 45 bp deletion. Finally, the *Pcer_FM_2_462* intergenic sequence contained a *FaSt* element, which was present across all assayed *Prunus* species.

The *Pdul_TK_22_1866* sequence contains a *FaSt* element in the intergenic region, which is also present in the ‘Nonpareil’ genome (98.89% identity), but absent from the homologs of ‘Lauranne’ and ‘Texas’. All other *Prunus* species also lack this *FaSt* copy in their homologous regions. The *FaSt* element in *Pdul_TK_4_761* was present in the homologous gene of ‘Texas’ but absent from ‘Lauranne’ and all other *Prunus* species. Additionally, *Pdul_TK_22_901* contains a *FaSt* element in the intron 5 of a *DEAD-box ATP-dependent RNA helicase 52C-like* gene, which is missing in the genome sequences of the ‘Nonpareil’, ‘Lauranne’, and ‘Texas’ cultivars. The *FaSt* element in the 5′ UTR of the *inositol 2-dehydrogenase/D-chiro-inositol 3-dehydrogenase* gene of ‘Tuono’ (*Pdul_TU_3_544*) was present in the ‘Lauranne’ gene but absent in the genome sequences of ‘Nonpareil’ and ‘Texas’. The *Pdul_TU_33_342* sequence, a unique intergenic region containing a *FaSt* copy, could not be detected in any other almond cultivars or *Prunus* species.

The *Pdom_HR_33_1460* sequence with its *FaSt* copy is present in the *P. domestica* draft genome, but the *FaSt* element is absent in *P. salicina* and other *Prunus* species. Finally, the *FaSt* elements in the *Pdom_NT_22_334* intron and the *Pdom_EL_4_433* intergenic sequence were not detected in *P. domestica* or any other *Prunus* genomes.

Variations were also observed in sequences lacking *FaSt* elements. Compared to the ‘Montmorency’ genome sequence, *Pcer_KJ_4_965* exhibited a ~30 bp deletion in the 3′ UTR, while the UTRs of *P. armeniaca* and *P. persica* were more divergent. The *Pcer_KJ_4_662* sequence, which contains an intron of a *peroxidase N1-like* gene, showed annealing of the FaSt-R primer to a degenerate region not part of a *FaSt* element. The complete sequence was found in the sour and sweet cherry, *P. fruticosa*, *P. yedonensis*, *P. campanulata*, and *P. speciosa* genomes. In contrast, the homologous sequences in plums, *P. mume*, *P. armeniaca*, *P. humilis*, *P. sibirica*, and *P. zhengheensis* exhibited a short (24 bp) deletion, while *P. persica*, *P. dulcis*, *P. kansuensis*, *P. davidiana*, *P. mandshurica*, *P. mongolica*, and *P. mira* contained a larger deletion of approximately 600 bp.

**Figure 7:**
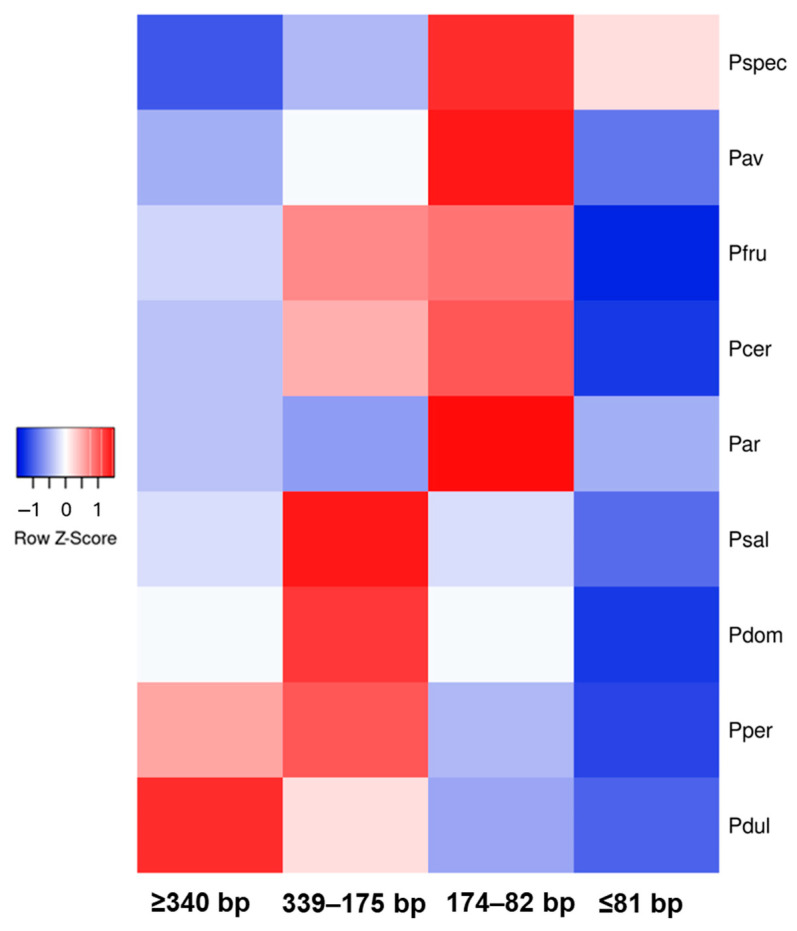
Heat map of in silico-identified *Falling Stones* (*FaSt*) transposon abundancy in nine *Prunus* genomes. The four size categories are as follows: almost complete (≥340 bp), 339–175 bp, 174–82 bp, and <82 bp. The colors represent genomic abundance, with red indicating high abundance and blue indicating low abundance. The columns correspond to the four size categories, and the rows represent the assayed genomes: *P. speciosa* (Pspec), *P. avium* (Pav), *P. fruticosa* (Pfru), *P. cerasus* (Pcer), *P. armeniaca* (Par), *P. salicina* (Psal), *P. domestica* (Pdom), *P. persica* (Pper), and *P. dulcis* (Pdul).

To compare the dynamics of *FaSt* accumulation in specific genomic regions with the broader genomic landscape of *FaSt* elements, the total number of *FaSt* copies was determined. We categorized the *FaSt* elements into four groups based on size: complete *FaSt* elements (≥340 bp), fragments exceeding 50% of the original length (339–175 bp), those between 174 and 82 bp, and those under 82 bp. Figure 7 illustrates the uneven distribution of *FaSt* elements of different sizes across the nine assayed genomes. *P. persica* and *P. dulcis* contained the largest number of full-length *FaSt* copies, while the 339–175 bp category was predominant in plum species. Species within the *Cerasus* subgenus exhibited the highest levels of truncated *FaSt* copies, ranging between 174 and 82 bp. *P. fruticosa* (1154) and *P. cerasus* (1594) had more full-length copies than *P. avium* (503) and *P. speciosa* (514). Similarly, *P. domestica* (1400) contained significantly more full-length *FaSt* copies than *P. salicina* (427). Interestingly, *P. armeniaca* displayed a *FaSt* profile more similar to that of the *Cerasus* species, with a higher proportion of fragmented *FaSt* copies compared to those in the *Prunus* subgenus.

## 3. Discussion

### 3.1. The SCoT Marker Efficiency in Prunus Is Increased by a FaSt Specific Assay

SCoT markers are designed to anneal to conserved regions flanking the start codon in plant genes [20] and have been successfully used in various plant species, including *Prunus* fruit trees [21,23,28,38,39]. The NDB and the average NDB per marker were consistent for both SCoT-only and SCoT–FaSt combinations, aligning with values reported in previous studies on *P. armeniaca* [39] and *P. sibirica* [21]. The marker efficiency parameters, including the average PIC and the collective *Rp* and genetic diversity measures (*h* and *I*), were not diminished by the addition of FaSt-R primers. On the contrary, the inclusion of FaSt-R primers in the SCoT–FaSt combinations resulted in enhanced levels of polymorphism. While the SCoT markers in this study produced a slightly lower percentage of polymorphic bands compared to other *Prunus* assays [21,39], the incorporation of FaSt-R significantly increased the observed polymorphism. The PIC values for dominant markers range from 0 to 0.5, and all assayed markers and their combinations exhibited higher PIC values than those reported for SCoT analysis in Chinese apricots [39].

TNB was lower, while NDB was slightly higher for the SCoT–FaSt combinations compared to the SCoT-only analyses. The fewer bands detected across a broader range of size categories spanned amplicon sizes from 150 to 8000 bp. This extended range included both smaller and larger fragments than those amplified by SCoT primers alone in several other plants, such as *Citrus*, *Punica*, and *Simmondsia* species [40,41,42]. The inclusion of FaSt-R primers reduced the gel pattern density, facilitating the more reliable identification of differentially expressed fragments. FaSt-R was specifically designed to anneal within the unique loop sequence of the non-autonomous transposon [35], enabling the detection of complete or partial *FaSt* copies within the *Prunus* genome. This specificity significantly reduces the number of amplicons, especially considering that the peach genome contains approximately 28,000 protein-coding genes [27] and around 1000 *FaSt* copies. The observed decrease in TNB and the changes in band sizes suggest that FaSt-R primers enhance the specificity of the PCR reaction. As expected, amplification using only SCoT primers will be less intense when FaSt-R is also available (Figure 1D). Additionally, SCoT primers can sometimes amplify gene fragments from pathogenic organisms infecting the host plant [43,44]. However, by incorporating FaSt-R, which selectively anneals to a *Prunus*-specific transposon, non-target amplicons that could interfere with the genetic analysis of crop plants are effectively excluded.

The SCoT–FaSt amplified fragments were used to conduct phylogenetic analyses, resulting in the construction of five subclusters in the neighbor-joining (NJ) tree, all with 99–100% bootstrap support. These subclusters included *P. persica*, *P. dulcis*, *P. domestica*, *P. armeniaca*, and cherry species, with *P. persica* and *P. dulcis*, as well as *P. avium* and *P. cerasus*, being relatively closer to each other. Notably, ‘*Feketicsi meggy’* was not grouped with ‘*Kántorjánosi 3*’, providing further evidence of its putative interspecific origin [45]. These phylogenetic relationships align closely with the divergence times of the respective species [6,11], further confirming that the SCoT–FaSt marker combinations generate robust phylogenetic signals that reliably reconstruct evolutionary relationships among species. It is even more accurate compared to the SCoT-only analysis, where the close evolutionary relationship between *P. persica* and *P. dulcis* [10,11,12] was not resolved.

A more detailed analysis of 28 *P. domestica* cultivars revealed five statistically supported clusters. The grouping of these cultivars reflected their origin and pedigree. Cluster BI, which received full support, included German cultivars from two distinct breeding programs—Hohenheim and Geisenheim. These cultivars shared a similar set of parental genotypes, including ‘Cacanska najbolja’, ‘Auerbach’, and ‘President’ [46]. Cluster BII, supported by a 90% bootstrap value, grouped the half-siblings of ‘Stanley’ [46]. Cluster BIII consisted of landraces of Hungarian origin, sister to a separate, statistically supported cluster for ‘Nemtudom’ and ‘Besztercei’ plums. Although the origin of these Hungarian plums remains unclear, they have evolved in specific geographic regions. Their distinct separation from other landraces was also observed in SSR analyses and chloroplast DNA sequence variation studies [16,17].

Cluster BV includes old cultivars from various countries, none of which have reported parentage. Another statistically unsupported cluster comprised Russian and Romanian cultivars, along with ‘Czar’. It is known that Romanian plum breeding programs in the 1960s and 1970s frequently used ‘Czar’ as a donor to introduce early ripening phenotype [47]. ‘Czar’ is the first European plum cultivar created in England in 1843, named after the Russian emperor who visited England [48]. It is therefore likely that accessions of this cultivar made their way into Russian breeding programs. The PCA plot provided a clearer representation of the four major groups. One group contained the two Hungarian landraces, which were distinctly separated from the other Hungarian plums, while cultivars from clusters BI and BII were much closer to each other. Our results indicate that SCoT–FaSt marker analysis provides sufficient phylogenetic signals to reliably reconstruct genetic relationships both among and within species.

Approximately one-third of the amplified fragments were smaller than 1 kb in size. Small fragment sizes have also been observed in SCoT analyses of species such as *Citrus* [40], *Simmondsia* [41], *Punica* [42], and many others. According to the original concept of the SCoT marker strategy, this would only be possible if two protein-coding genes are located in opposite directions but very close to each other. To verify whether many genes in the *Prunus* genome are indeed this closely located, and to ensure that the resulting fragments are the product of joint amplification by the SCoT and FaSt-R primer pairs, we cloned several fragments and determined their DNA sequences.

### 3.2. Functional and Evolutionary Aspects of the Genomic Landscape of FaSt

Each cloned amplicon was flanked by 18 and 22 bp sequences corresponding to the SCoT and FaSt-R primers at their respective ends, confirming that all randomly selected fragments were indeed amplified by a combination of these oligonucleotides. This supports our hypothesis that fragments amplified by the sequence-specific FaSt-R primer predominate among the amplicons. However, six of the sequences contained only the FaSt-R primer sequence and lacked the expected ~230 bp segment of the *FaSt* element. This suggests that while the FaSt-R primer anneals specifically, its target site is more frequent in the genome than the complete *FaSt* elements, which leads to the amplification of more fragments across all combinations. These annealing sites may have arisen through the accumulation of base substitutions outside the *FaSt* element or internal deletions within the *FaSt*, as mutations in a MITE are unlikely to significantly affect fitness [49,50].

The vast majority (26) of the sequences contained an identifiable *FaSt* element, with 11 of them inserted in non-coding regions of protein-coding genes, including UTRs and introns. This aligns with the genome-wide distribution of MITEs in many species, which shows a bias toward UTRs and introns, with many more copies present in these regions than in exons [35,51,52]. The remaining 15 *FaSt* elements were detected in intergenic regions, consistent with the known insertion preference of *FaSt* in AT-rich sequences [35]. SCoT markers were designed to anneal to the conserved region around the ATG start codon of plant genes [20]. The size of the sequences ranged from 251 to 1866 bp, which is consistent with amplicon size ranges reported in other studies using SCoT analysis [40,41,42]. This makes it unlikely that SCoT primers amplified regions between protein-coding genes in opposite orientations. Very few studies have reported sequence data from SCoT-amplified fragments to verify the annealing of SCoT primers to the start codon. A brief survey of sequences used to design SCAR markers (as listed by Rai [38]) confirmed that most did not include the sequences surrounding the start codon. Our partial sequences of 17 protein-coding genes verified that SCoT primers annealed to exonic regions outside the start codon. Plant introns are AU-rich, in contrast to GC-rich exons [53,54], and the higher GC content of exons is crucial for the increased stability of RNA structures, including mRNA and rRNA [55,56]. This explains why many SCoT–FaSt primer combinations amplified parts of protein and RNA-coding genes. Non-genic segments of the genome exhibit variable levels of GC content [56], which could explain why some SCoT primers annealed to these regions. These results suggest that under the PCR conditions recommended for SCoT analysis [20], the primers do not selectively bind to the start codon regions but rather show a tendency to anneal within the GC-rich segments of the genome, which nonetheless creates a good chance of finding genes.

*FaSt* copies were identified in the intron and 5′ and 3′ UTRs of several genes, some of which are involved in key physiological processes. A *FaSt* was found in the intron of five genes, including the sour cherry *BTB/POZ domain-containing protein At4g08455-like* and *pectinesterase* genes. BTB/POZ proteins are associated with growth and development through mechanisms such as ethylene biosynthesis, disease resistance, and hormone perception, which occur via selective ubiquitination and kinase activation [57]. Pectinesterases are involved in pectin degradation, thereby accelerating the rate of fruit softening [58]. Additionally, two *P. domestica* genes were found to contain *FaSt* insertions in their introns. The glycosyltransferase BC10-like proteins are involved in metabolic processes and play a role in fruit ripening and responses to abiotic stresses [59], while the leaf rust 10 disease-resistance locus receptor-like protein kinase-like proteins, members of LRR-NBS resistance proteins, contribute to enhanced resistance to *Xanthomonas* [60]. In *P. dulcis*, the *DEAD-box ATP-dependent RNA helicase*-encoded proteins suppress potyvirus accumulation through an interaction with the viral protein VPg, which is essential for viral infection [61].

A *FaSt* copy was identified in the 5′ UTR of the plum *polygalacturonase ADPG1* gene, whose protein product is involved in pectin degradation and fruit softening [62]. The 3′ UTRs of the *P. domestica ethylene-responsive transcription factor ERF053-like* and *beta-D-glucosyl crocetin beta-1,6-glucosyltransferase* genes also contained *FaSt* copies. Ethylene response factor (ERF) transcription factors regulate fruit ripening as well as resistance to biotic and abiotic stresses [63], while the beta-D-glucosyl crocetin beta-1,6-glucosyltransferase enzyme participates in the flavonoid and anthocyanin biosynthetic pathways [64]. Since sequence alterations, including MITE insertions in UTRs and introns, have frequently been associated with regulatory roles [65,66,67], further studies are needed to determine whether the presence of *FaSt* elements affects the expression of these genes.

The identification of genomic regions homologous to our SCoT–FaSt sequences in other *Prunus* genomes has provided further evolutionary insights. We found a *FaSt* element present in one *P. avium* and three *P. cerasus* sequences, which was absent from the homologous genomic regions in all other species within the *Prunus* subgenus. Two of those (*Pav_CG_3_910* and *Pcer_KJ_2_343*) also lacked the *FaSt* element in *P. fruticosa*, and the *FaSt* in *Pcer_KJ_4_1000* was missing from *P.* × *kanzakura*. The phylogenetic distribution of such *FaSt* copies helped determine their relative insertion times. The *FaSt* in *Pcer_FM_2_462* was present in all species, indicating that it was inserted between 47 and 52 Mya according to TimeTree 5 estimations based on data from Chin et al. [6], Guo et al. [68], and Pouget et al. [69]. The *FaSt* in *Pcer_FM_4_496* was found in all species of the *Cerasus* subgenus but absent in all other *Prunus* species, suggesting that it emerged around 47 Mya. Three *FaSt* elements were inserted approx. 47–43 Mya on the phylogenetic lineage of *P. avium* and *P. cerasus*. Additionally, the presence and size of other indels in *Pcer_KJ_4_1000* and *Pcer_KJ_4_662* also followed the known speciation events in the *Prunus* genus [6]. The uneven distribution of certain *FaSt* elements between the *Cerasus* and *Prunus* subgenera prompted us to consider whether a burst of *FaSt* insertions around 47–43 Mya was a significant evolutionary event. To investigate this, we analyzed the proportion of complete and partial *FaSt* elements in the genomes of several species within both subgenera. Our analysis revealed an unequal distribution of *FaSt* elements of varying sizes, with species in the *Cerasus* subgenus predominantly harboring fragmented *FaSt* copies (smaller than the half of the 349 bp *FaSt*), while most species in the *Prunus* subgenus contained a significantly higher number of complete *FaSt* elements. The only exception was *P. armeniaca*, which contained many partial *FaSt* elements, though complete and presumably active *FaSt* copies were also identified in this species [35,36]. Polyploid species exhibited two to three times more *FaSt* copies. Overall, our findings suggest that many of the *FaSt* elements of *P. avium* and *P. cerasus* are older than those of *P. persica*, *P. dulcis*, and *P. domestica*. A sudden burst of *FaSt* insertions in one of the phylogenetic lineages may have resulted in an accelerated differentiation. MITE insertions have been associated with increased capacity for unequal crossing over [70], alternative splicing [71], and modifications to gene regulation [72]. Similar evolutionary events have been observed in primates following a sudden increase in Alu repeats around 40 Mya [73].

The differential presence of *FaSt* copies in almond sequences was fully consistent with reported connections between germplasm and the pedigree of tested cultivars. *Pdul_TK_22_1866* and *Pdul_TK_4_761* contained *FaSt* elements also found in ‘Nonpareil’ and ‘Texas’, respectively. It is well documented that Hungarian almond breeding programs have utilized accessions from the USA, which is reflected in the self-incompatibility genotypes of the cultivars [74]. The *FaSt* element in the ‘Tuono’ *Pdul_TU_3_544* sequence was also present in ‘Lauranne’, which aligns with ‘Lauranne’ being an offspring of the cross ‘Ferragnés’ × ‘Tuono’ [75]. Two unique *FaSt* copies in the ‘Tuono’ (*Pdul_TU_33_342*) and ‘Tétényi keményhéjú’ (*Pdul_TK_22_901*) sequences suggest recent *FaSt* insertions, as confirmed by the high proportion of complete *FaSt* elements in the *P. dulcis* genome (Figure 7) and consistent with the activity of transposable elements in almond [12]. Recent *FaSt* insertions were also seen in the sequences of hexaploid *P. domestica*.

The combination of SCoT primers and a newly designed sequence-specific primer targeting the *Prunus* non-autonomous transposon, *FaSt*, provided a molecular marker with enhanced polymorphism detection, free from non-target amplification, and capable of delivering reliable phylogenetic information at both inter- and intraspecific taxonomic levels. The identification of variations in the genome-wide distribution of *FaSt* insertions enabled the detection of several genes and genomic regions exhibiting insertional polymorphisms. A more detailed analysis of these variations could provide valuable insights into the genetic diversity of breeding germplasm, the reconstruction of evolutionary relationships, and the genome-shaping impact of an active Mutator-type MITE in the *Prunus* genome.

## 4. Materials and Methods

### 4.1. Plant Material and DNA Isolation

Two sets of experiments were carried out, with the first on two cultivars of six *Prunus* species, *P. armeniaca* L. (apricot), *P. dulcis* (Mill.) D.A. Webb (almond), *P. avium* L. (sweet cherry), *P. persica* (L.) Batsch (peach), *P. cerasus* L. (sour cherry), and *P. domestica* L. (European plum). In the second experiment, 28 cultivars of European plum were analyzed; all cultivars are listed in Table 2. The trees were sampled in the *Prunus* germplasm collection of the Department of Plant Biotechnology, Hungarian University of Agriculture and Life Sciences (MATE, Budapest, Hungary). Total genomic DNA was isolated from buds using a DNeasy Plant Mini Kit (Qiagen, Hilden, Germany) according to the manufacturer’s instructions.⁠ The quantity and quality of DNA were analyzed by NanoDrop^TM^ ND-1000 spectrophotometer (Bio-Science, Budapest, Hungary).

### 4.2. Genomic PCR with SCoT and FaSt-Specific Primers

For genomic PCR, 19 (1, 2, 4–7, 11–13, 16, 18, 19, 21–24, 27, 32 and 34) and 17 (1–5, 7–11, 15, 16, 19, 21–23, and 34) SCoT primers were used for two cultivars each of six *Prunus* species and 28 *P. domestica* cultivars, respectively. A new primer, FaSt-R (5′-TCTTAGAAATTACAAAACTACC-3′) was designed to anneal to a unique sequence of the *Falling Stone* MITE. The FaSt-R and all SCoT primers [20] were used separately and combined with each other. Approximately 40–70 ng of genomic DNA was used for PCR amplification in a 12 µL reaction volume, containing 10 × DreamTaqTM Green buffer (Thermo Fisher Scientific, Waltham, MA, USA) with final concentrations of 1.5 mM MgCl_2_, 0.2 mM of dNTPs, 0.4 µM of the adequate primers, and 0.625 U of DreamTaqTM DNA polymerase (Thermo Fisher Scientific, Waltham, MA, USA). The PCR amplification was carried out in a 27Swift MaxPro thermocycler (ESCO Healthcare, Singapore, Republic of Singapore). Amplification was run at 94 °C for 5 min, followed by 40 cycles of denaturation at 94 °C for 1 min, primer annealing at 50 °C for 1 min, and elongation at 72 °C for 2 min; the final extension was 10 min at 72 °C. Amplicons were detected by 1.2% TBE agarose gels stained with ethidium bromide (EtBr) at 80 V for 3 h. Fragment sizes were estimated by comparison with the GeneRuler™ 1-kb DNA Ladder (Thermo Fisher Scientific, Waltham, MA, USA).

### 4.3. Cloning and DNA Sequencing

Direct cloning of PCR products was carried out using the InsTAclone PCR Cloning Kit (Thermo Fisher Scientific, Waltham, MA, USA) and JM109 competent cells, isolated with a GeneJET^TM^ Plasmid Miniprep Kit (Thermo Fisher Scientific, Waltham, MA, USA). Nucleotide sequences of 3 samples were determined for each fragment in both directions using M13 sequencing primers in an ABI 3500 XL Genetic Analyzer (Applied Biosystems, Foster City, CA, USA).

### 4.4. Data Evaluation and Bioinformatics Analyses

The banding patterns of the SCoT, FaSt and SCoT–FaSt marker analyses were scored as binary data with strong and clearly separated bands as present (1) and absent bands as (0); weak or ambiguous bands were excluded. The TNB, NDB, SRA, PA ≤ 1 kb, and PPB were calculated in MS Excel, while PIC, *h*, *I*, and *Rp* were determined using the iMEC, https://irscope.shinyapps.io/iMEC/ (accessed on 11–16 January 2025) [77] and Popgene 1.32 [78] programs. A genetic similarity matrix was calculated using Jaccard’s index, which is appropriate for binary presence/absence data. Based on this matrix, a principal component analysis (PCA) was performed, and a phylogenetic tree was constructed using the neighbor-joining (NJ) method in PAST v4.03 [79]. Bootstrap analysis with 1000 replicates was carried out to assess the robustness of the tree, and the resulting support values are indicated on the branches. The BLASTn analysis at the NCBI and the available genome sequences of the *Prunus* species at the GDR, https://www.rosaceae.org/blast (accessed on 20–30 January 2025) databases were used for homology searches [80]. The BLASTn 2.12.0+ analysis at GDR [81] was carried out using *P. cerasus* ‘Montmorency’ v1.0.a2, *P. avium* ‘Tieton’ genome v2.0, *P. fruticosa* 27e12(2) v1.0, *P. dulcis* ‘Lauranne’ v1.0.a1, ‘Nonpareil’ v1.0, ‘Texas’ v3.0, *P. armeniaca* ‘Stella’ v1.0, *P. persica* Genome V2.0.a1, *P. yedoensis* Genome v1.0, *P. domestica* Draft Genome v1.0 genomes, *P. campanulata* v1.0, *P. kansuensis* genome v2.0, *P. mume* Nanko v1.0 genome, *P. salicina* Sanyueli genome v2.0, *Prunus speciosa* IZO01 v1.0, *P. davidiana* Genome v2.0, *P. mandshurica* genome v1.0, *P. mongolica* v1.0, *P. mira* Genome v2.0, *P. sibirica* CH320_5 genome v1.0, and *P. zhengheensis* v1.0. Sequences were aligned and presented with MEGA 7 [82] and BioEdit v. 7.2.0. [83], respectively. The phylogenetic tree of 12 *Prunus* and a *Malus* species was built based on the divergence times determined by Chin et al. [6] and by using the TimeTree 5 [83] database (https://timetree.org, accessed on 14 February 2025). The *FaSt* sequence [35] was used for BLASTn analysis on the *P. speciosa*, *P. avium*, *P. fruticosa*, *P. cerasus*, *P. armeniaca*, *P. salicina*, *P. domestica*, *P. persica*, and *P. dulcis* genomes. The hits were arranged in four size categories (≥340 bp, 339–175 bp, 174–82 bp, and ≤82 bp) and their genomic copy numbers were determined in MS Excel. A heatmap was generated for the frequency of complete and partial *FaSt* elements in the analyzed *Prunus* genomes using the expression function within Heatmapper [84].

## Figures and Tables

**Table 1:** Summary of statistical parameters obtained from 19 SCoT primers, the FaSt-R primer, and their combinations across 12 cultivars representing six *Prunus* species.

	TNB *	NDB *	SRA *	PA ≤ 1 kb *	PPB *	PIC *	*Rp* *	*h* *	*I* *
SCoT 1	73	11	350–2200	43.8	83.6	0.37	6.17	0.35	0.51
SCoT 2	88	14	650–3000	6.8	59.1	0.28	6.00	0.24	0.37
SCoT 4	133	23	250–4000	59.4	72.9	0.35	12.50	0.28	0.43
SCoT 5	86	14	500–3000	47.7	86.0	0.37	8.33	0.33	0.50
SCoT 6	81	12	350–3000	55.6	85.2	0.33	5.83	0.30	0.45
SCoT 7	35	8	1100–3500	0.0	100.0	0.37	4.50	0.29	0.46
SCoT 11	51	7	2000–4500	0.0	52.9	0.33	3.83	0.24	0.37
SCoT 12	70	11	350–3000	60.0	100.0	0.41	8.00	0.35	0.53
SCoT 13	98	16	300–4500	36.7	100.0	0.38	11.00	0.33	0.50
SCoT 16	76	10	750–2250	47.6	68.4	0.34	5.33	0.32	0.47
SCoT 18	119	23	300–2500	67.2	69.7	0.37	12.83	0.28	0.44
SCoT 19	82	14	500–3000	23.2	85.4	0.36	7.33	0.32	0.48
SCoT 21	78	12	500–2500	21.8	84.6	0.37	6.67	0.34	0.51
SCoT 22	79	12	800–3000	27.8	54.4	0.33	6.50	0.25	0.39
SCoT 23	54	10	400–4000	27.5	33.3	0.23	3.00	0.26	0.32
SCoT 24	72	11	500–2000	54.2	100.0	0.38	6.67	0.38	0.56
SCoT 27	90	14	300–6000	64.4	73.3	0.37	7.17	0.29	0.44
SCoT 32	70	12	350–3000	40.0	82.9	0.34	6.00	0.31	0.46
SCoT 34	63	12	650–4000	17.5	81.0	0.39	7.83	0.30	0.46
Mean	78.8	12.9		36.9	77.5	0.35	7.13	0.30	0.46
FaSt-R	46	10	700–2500	34.8	73.9	0.36	5.67	0.25	0.40
SCoT 1 + FaSt-R	73	10	600–4000	32.9	100.0	0.35	6.17	0.35	0.52
SCoT 2 + FaSt-R	74	10	300–4000	27.0	83.8	0.35	5.67	0.36	0.53
SCoT 4 + FaSt-R	49	11	450–3000	36.7	100.0	0.34	5.50	0.29	0.45
SCoT 5 + FaSt-R	77	11	300–8000	6.5	84.4	0.28	4.50	0.30	0.46
SCoT 6 + FaSt-R	65	14	150–3500	47.7	81.5	0.32	6.50	0.26	0.41
SCoT 7 + FaSt-R	30	9	300–4000	30.0	100.0	0.35	4.67	0.24	0.39
SCoT 11 + FaSt-R	112	21	250–4000	33.9	78.6	0.38	13.00	0.29	0.45
SCoT 12 + FaSt-R	56	10	450–8000	46.4	100.0	0.42	6.50	0.34	0.52
SCoT 13 + FaSt-R	84	13	700–5000	41.7	85.7	0.35	7.00	0.34	0.50
SCoT 16 + FaSt-R	67	16	480–8000	32.8	100.0	0.38	8.83	0.29	0.45
SCoT 18 + FaSt-R	86	14	500–5500	11.6	84.8	0.32	6.33	0.30	0.46
SCoT 19 + FaSt-R	91	19	290–6000	36.3	86.8	0.37	10.50	0.29	0.44
SCoT 21 + FaSt-R	75	13	150–6000	42.7	100.0	0.35	6.83	0.34	0.51
SCoT 22 + FaSt-R	94	16	750–6000	34.0	87.2	0.36	8.67	0.32	0.48
SCoT 23 + FaSt-R	70	13	750–4500	20.0	91.4	0.38	6.33	0.26	0.42
SCoT 24 + FaSt-R	54	10	250–4000	37.0	55.6	0.31	5.00	0.22	0.35
SCoT 27 + FaSt-R	31	12	180–3500	45.2	100.0	0.34	4.83	0.22	0.37
SCoT 32 + FaSt-R	50	10	400–6000	20.0	100.0	0.38	5.33	0.32	0.49
SCoT 34 + FaSt-R	45	16	280–3000	44.4	100.0	0.30	5.83	0.20	0.34
Mean	67.5	13.1		33.0	90.5	0.35	6.74	0.29	0.45

* TNB, total number of bands; NDB, number of differently sized bands; SRA, size range of amplicons; PA ≤ 1 kb, percentage of amplicons with size ≤ 1000 bp; PPB, percentage of the polymorphic bands; PIC, polymorphism information content; *Rp*, resolving power; *h*, Nei’s gene diversity; *I*, Shannon’s information index.

**Table 2:** Origin of *Prunus* cultivars used in the study [17,76].

Cultivar Name	Species	Pedigree (Year of Cultivar Recognition)	Country of Origin
‘Besztercei Bt2’	*P. domestica*	Clone of Besztercei (1974)	Hungary
‘Bühler Frühzwetschge’	*P. domestica*	German landrace (1840)	Germany
‘Canada giant’ (‘Sumgita’)	*P. avium*	Van × Sam (1992)	Canada
‘Ceglédi óriás’	*P. armeniaca*	Local selection, Izsák (1953)	Hungary
‘Collins’	*P. persica*	Jerseyland × NJ-188 (1955)	USA
‘Condor’	*P. persica*	Unknown	USA
‘Czar’	*P. domestica*	Prince Engelbert × Early Prolific (1874)	United Kingdom
‘Duránci’	*P. domestica*	Hungarian landrace (Borsod)	Hungary
‘Elena’	*P. domestica*	Fellenberg × Stanley (1993)	Germany
‘Empress’	*P. domestica*	Unknown (2000)	Italy
‘Fehérszilva’	*P. domestica*	Hungarian landrace (Duna-Tisza-köz, Borsod, Bácska) (2014)	Hungary
‘Feketicsi meggy’	*P. cerasus*	Landrace, Bácsfeketehegy	Serbia
‘Giant’	*P. domestica*	Ageni × Pond’s Seedling (1893)	USA
‘Gönci magyar kajszi’	*P. armeniaca*	Selected clone of Hungarian Best (1960)	Hungary
‘Grand prize’	*P. domestica*	Unknown seedling of Burbank (1937)	USA
‘Haganta’	*P. domestica*	Cacanska najbolja× Valor (2003)	Germany
‘Hanita’	*P. domestica*	President × Auerbacher (1980)	Germany
‘Haroma’ *	*P. domestica*	(Ortenauer × Stanley 34) × Hanita (1993)	Germany
‘Jojo’	*P. domestica*	Ortenauer × Stanley (1991)	Germany
‘Kántorjánosi 3’	*P. cerasus*	Selected clone of a landrace, Kántorjános (1994)	Hungary
‘Katalin’	*P. avium*	Germersdorfer × Podyebrad (1989)	Hungary
‘Nemtudom P3’	*P. domestica*	Selected clone of a landrace (2012)	Hungary
‘Opal’	*P. domestica*	Early Favourite × Oullins gage (1925)	Sweden
‘Páczelt szilvája’	*P. domestica*	Unknown, Páczelt János, Nagykároly (end of the 19th century)	Hungary
‘Presenta’	*P. domestica*	President × Ortenauer (1996)	Germany
‘Sanctus Hubertus’	*P. domestica*	Mater Dolorosa × Early Rues (1963)	Belgium
Soviet 2	*P. domestica*	Soviet seedling	Russia
Soviet 3	*P. domestica*	Soviet seedling	Russia
‘Tétényi keményhéjú’	*P. dulcis*	Open pollination from Burbank magonca (1980)	Hungary
‘Topend plus’	*P. domestica*	Cacanska najbolja × Valor (1994)	Germany
‘Topfive’	*P. domestica*	Cacanska najbolja × Bühler Frühzwetsche (1999)	Germany
‘Tophit’	*P. domestica*	Cacanska najbolja × President (1988)	Germany
‘Toptaste’ *	*P. domestica*	Valor × Hauszwetschke (1994)	Germany
‘Tuleu timpuriu’	*P. domestica*	Tuleu gras × Sermina (1967)	Romania
‘Tuono’	*P. dulcis*	Unknown (~1830)	Italy
‘Vengerka virla’	*P. domestica*	Unknown	Russia
‘Vörös nagygyümölcsű’	*P. domestica*	Landrace	Hungary
‘Wagenheim’	*P. domestica*	Unknown (1837)	Germany

* *P. domestica* cultivars included in both sets of experiments.

## Data Availability

A total of 32 sequences were submitted to the NCBI GenBank database under the accession numbers PQ869779–PQ869809.

## Supplementary Materials

The following supporting information can be downloaded at https://www.mdpi.com/article/10.3390/ijms26093972/s1.

## Abbreviations

The following abbreviations are used in this manuscript:

AFLP	amplified fragment length polymorphism
BLASTn	Basic Local Alignment Search Tool
*FaSt*	*Falling Stones*
GDR	Genome Database of Rosaceae
*h*	Nei’s gene diversity
*I*	Shannon’s information index
iPBS	inter-primer binding site
ISSR	inter-simple sequence repeat
LTR	long terminal repeat retrotransposons
MITE	miniature inverted-repeat transposable elements
NCBI	National Center for Biotechnology Information
NDB	number of differently sized bands
PA ≤ 1 kb	percentage of amplicons with size ≤ 1000 bp
PIC	polymorphism information content
PPB	percentage of the polymorphic bands
RAPD	random amplified polymorphic DNA
*Rp*	resolving power
SCoT	start-codon-targeted
SRA	size range of amplicons
SSR	microsatellite
TE	transposable elements
TIR	terminal inverted repeats
TNB	total number of bands
TSD	target site duplications
