# Supplementary material for: The Combination of Start-Codon-Targeted (SCoT) and Falling Stone (FaSt) Transposon-Specific Primers Provides an Efficient Marker Strategy for Prunus Species
Source: Int J Mol Sci. 2025 Apr 23;26(9):3972. doi: 10.3390/ijms26093972 (PMC12071656; doi:10.3390/ijms26093972)
Supplement: Supplementary file 1 [file ijms-26-03972-s001.zip › Figure S1.pdf]

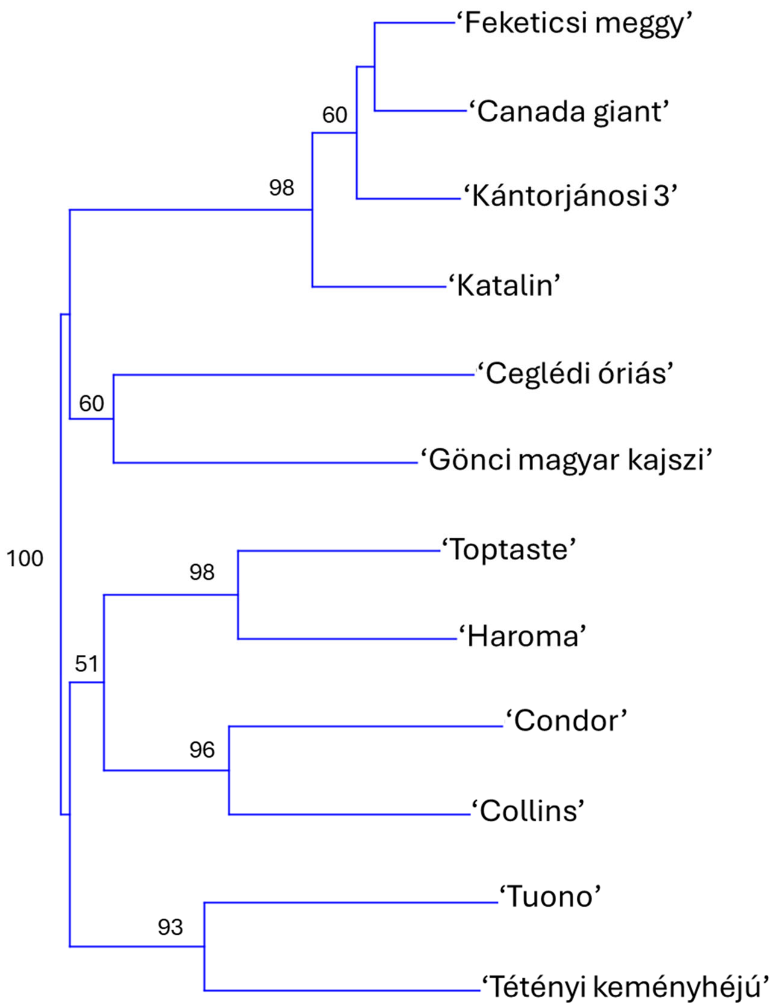

Figure S1: Phylogenetic analysis of *Prunus* based on data from 19 SCoT primer assays using the neighbor-joining method and Jaccard’s similarity coefficients. Twelve cultivars representing six *Prunus* species: *P. dulcis*, *P. armeniaca*, *P. domestica*, *P. persica*, and members of the *Cerasus* subgenus (*P. avium* and *P. cerasus*).
