# Supplementary material for: The Combination of Start-Codon-Targeted (SCoT) and Falling Stone (FaSt) Transposon-Specific Primers Provides an Efficient Marker Strategy for Prunus Species
Source: Int J Mol Sci. 2025 Apr 23;26(9):3972. doi: 10.3390/ijms26093972 (PMC12071656; doi:10.3390/ijms26093972)
Supplement: Supplementary file 1 [file ijms-26-03972-s001.zip › Figure S2.pdf]

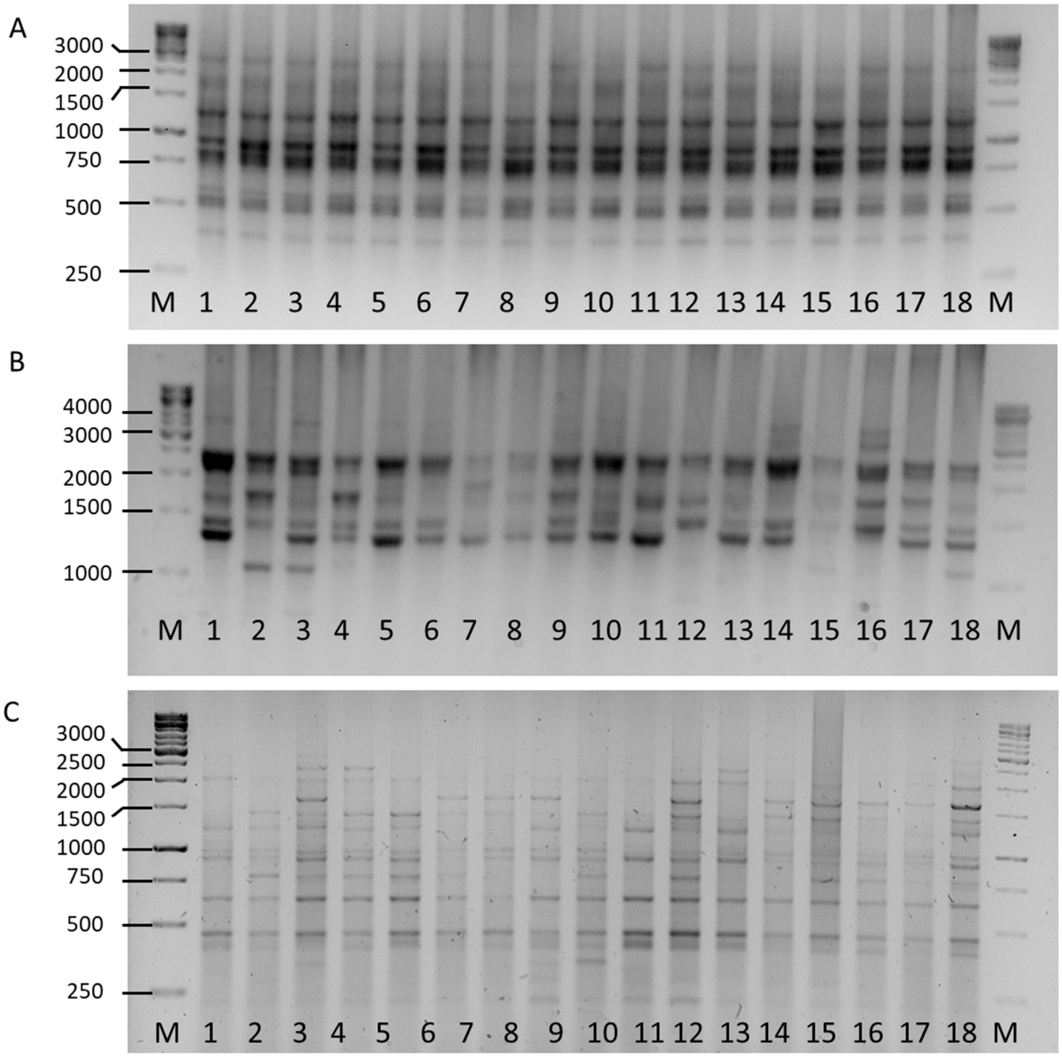

Figure S2: Patterns of amplified fragments in PCR of European plum (*Prunus domestica*) cultivars using the SCoT1 (A), FaSt-R (B) and a combination of SCoT1 and FaSt-R (C) primers. Labels are the followings: (M) GeneRuler 1 kb DNA ladder, (1) 'Bühler Frühzwetschge', (2) 'Czar', (3) 'Grand Prize', (4) 'Fehérszilva', (5) 'Páczelt szilvája', (6) Soviet 3, (7) 'Tuleu timpuriu', (8) 'Vengerka virla', (9) 'Vörös nagygyümölcsű', (10) 'Besztercei Bt.2', (11) 'Sanctus Hubertus', (12) 'Wagenheim', (13) 'Giant', (14) Soviet 2, (15) 'Duránci', (16) 'Opal', (17) 'Haganta' and (18) 'Presenta'.
