# Supplementary material for: The Combination of Start-Codon-Targeted (SCoT) and Falling Stone (FaSt) Transposon-Specific Primers Provides an Efficient Marker Strategy for Prunus Species
Source: Int J Mol Sci. 2025 Apr 23;26(9):3972. doi: 10.3390/ijms26093972 (PMC12071656; doi:10.3390/ijms26093972)
Supplement: Supplementary file 1 [file ijms-26-03972-s001.zip › Table S1.pdf]

Table S1. Accession numbers and labels of the sequenced amplicons from the SCoT-FaSt-R PCR assays with amplicon size, parameters of BlastN homology searches and identified regions.

**Accession Nu.**	**Size bp**	**BLAST homolog**	**Accession Nu. of BLAST hit**	**Coverage %**	***E* value**	**Identity %**	**Regions (bp)**
SCoT1-FaSt-R, *Prunus domestica* cultivar ‘Topfive’							
PQ869779<br>*Pdom 1-TF-907*	907	*P. mume polygalacturonase ADPG1* (LOC103339337), mRNA	XM_008242608.2	77	0.0	96.13	5’ UTR <1..293<br>*FaSt* <1..211<br>exon 294..>907
SCoT1-FaSt-R, *Prunus domestica* cultivar ‘Besztercei Bt.2’							
PQ869780<br>*Pdom_BB_1_908*	908	*P. mume polygalacturonase ADPG1* (LOC103339337), mRNA	XM_008242608.2	77	0.0	96.14	5’ UTR <1..293<br>*FaSt* <1..211<br>exon 294..>908
SCoT2-FaSt-R, *Prunus cerasus* cultivar ‘Kántorjánosi 3’							
PQ869781<br>*Pcer_KJ_2_1227*	1,227	*P. avium beta-D-glucosyl crocetin beta-1,6-glucosyltransferase-like* (LOC110763544), mRNA	XM_021966336	100	0.0	99.92	exon <1..1101<br>3’ UTR 1102..>1227
SCoT2-FaSt-R, *Prunus cerasus* cultivar ‘Feketicsi meggy’							
PQ869782<br>*Pcer_FM_2_1227*	1,227	*P. avium beta-D-glucosyl crocetin beta-1,6-glucosyltransferase-like* (LOC110763544), mRNA	XM_021966336.1	100	0.0	99.84	exon <1..1101<br>3’ UTR 1102..>1227
SCoT2-FaSt-R, *Prunus cerasus* cultivar ‘Kántorjánosi 3’							
PQ869783<br>*Pcer_KJ_2_343*	343	*P. armeniaca genome assembly, chromosome: 1*	OZ046360.2	48	1e-55	91.62	*FaSt* <1..>166<br>Intergenic 167.. >343
SCoT2-FaSt-R, *Prunus cerasus* cultivar ‘Feketicsi meggy’							
PQ869784<br>*Pcer_FM_2_462*	462	*P. armeniaca genome assembly, chromosome: 2*	OZ046361.2	100	0.0	96.98	Intergenic <1..263<br>*FaSt* <264..>462
SCoT3-FaSt-R, *Prunus dulcis* cultivar ‘Tuono’							
PQ869785<br>*Pdul_TU_3_544*	544	*P. dulcis inositol 2-dehydrogenase/D-chiro-inositol 3-dehydrogenase* (LOC117628739)	XM_034361251.1	48	4e-132	100	5’ UTR <1..444<br>*FaSt* <1..211<br>exon 445..>544

**Accession Nu.**	**Size bp**	**BLAST homolog**	**Accession Nu. of BLAST hit**	**Coverage %**	***E* value**	**Identity %**	**Regions (bp)**
SCoT3-FaSt-R, *Prunus avium* cultivar ‘Canada giant’							
PQ869786<br>*Pav_CG_3_910*	910	*P. armeniaca genome assembly, chromosome: 8*	OZ046367.2	56	6e-153	94.93	*FaSt* <1..193<br>Intergenic 194.. >910
SCoT4-FaSt-R, *Prunus dulcis* cultivar ‘Tétényi keményhéjú’							
PQ869787<br>*Pdul_TK_4_761*	761	*Prunus persica* uncharacterized LOC18771123 (LOC18771123), transcript variant X2, mRNA	OZ022203.1	48	2e-157	94.57	5’ UTR <1..210<br>exon 211..>368<br>intron 369.. >761<br>*FaSt* 550.. .>761
SCoT4-FaSt-R, *Prunus cerasus* cultivar ‘Kántorjánosi 3’							
PQ869788<br>*Pcer_KJ_4_662*	662	*P. avium peroxidase N1-like* (LOC110751195), mRNA	XM_021951630.1	77	0.0	94.45	exon <1..>343<br>intron 344.. >662
SCoT4-FaSt-R, *Prunus cerasus* cultivar ‘Kántorjánosi 3’							
PQ869789<br>*Pcer_KJ_4_965*	965	Similar to *At1g77260* Probable *methyltransferase PMT10* (*Arabidopsis thaliana* OX=3702)	Pcer_083304-RA	100	0.0	99.00	exon <1..555<br>3’ UTR 556..>965
SCoT4-FaSt-R, *Prunus cerasus* cultivar ‘Kántorjánosi 3’							
PQ869790<br>*Pcer_KJ_4_1000*	1,000	*P. avium BTB/POZ domain-containing protein At4g08455-like* (LOC110766395), mRNA	XM_021969719.1	13	6e-59	100.00	*FaSt* <1..201<br>exon <871..>1000<br>intron <1.. >870
SCoT4-FaSt-R, *Prunus cerasus* cultivar ‘Feketicsi meggy’							
PQ869791<br>*Pcer_FM_4_1056*	1,056	*P. dulcis U1 spliceosomal RNA* (LOC117616929), ncRNA	XR_004584266.1	15	8e-68	96.91	intergenic <1..137<br>snRNA 138..299<br>intergenic 300..>1056<br>*FaSt* <862..>1056
SCoT4-FaSt-R, *Prunus cerasus* cultivar ‘Feketicsi meggy’							
PQ869792<br>*Pcer_FM_4_496*	496	*P. avium pectinesterase* (LOC110749177), mRNA	XM_021949211.1	23	3e-49	100.00	*FaSt* <1..202<br>exon <384..>496<br>intron <1..383
SCoT4-FaSt-R, *Prunus domestica* cultivar ‘Elena’							
PQ869793<br>*Pdom_EL_4_433*	433	*P. armeniaca genome assembly, chromosome: 8*	OZ046367.2	100	0.0	96.31	Intergenic <1..>220<br>*FaSt* <221..>433

**Accession Nu.**	**Size bp**	**BLAST homolog**	**Accession Nu. of BLAST hit**	**Coverage %**	***E* value**	**Identity %**	**Regions (bp)**
SCoT4-FaSt-R, *Prunus domestica* cultivar 'Elena'							
PQ869794<br>*Pdom_EL_4_766*	766	*P. mume 7-deoxyloganetin glucosyltransferase-like* (LOC103319662), mRNA	XM_008221234.2	100	0.0	98.56	exon <1..711<br>3' UTR 712..>766
SCoT16-FaSt-R, *Prunus persica* cultivar 'Collins'							
PQ869795<br>*Pper_CO_16_454*	454	*P. armeniaca genome assembly, chromosome: 2*	OZ046361.2	99	1e-87	92.44	intergenic <1..249<br>*FaSt* 250..>454
SCoT19-FaSt-R, *Prunus armeniaca* cultivar 'Goldrich'							
PQ869796<br>*Parm_GR_19_251*	251	*P. armeniaca genome assembly, chromosome: 4*	OZ046363.2	100	2e-107	95.62	intergenic <1..43<br>*FaSt* 44..>251
SCoT21-FaSt-R, *Prunus domestica* cultivar 'Toptaste'							
PQ869810<br>*Pdom_TT_21_269*	269	*P. persica WUSCHEL-related homeobox 9* (LOC18778807), transcript variant X2, mRNA	XM_020562726.1	48	5e-54	97.69	exon <1..>128<br>intron 129..>269
SCoT22-FaSt-R, *Prunus dulcis* cultivar 'Tétényi keményhéjú'							
PQ869797<br>*Pdul_TK_22_1866*	1,866	*P. dulcis genome assembly, chromosome: 3*	OZ022199.1	100	0.0	95.17	*FaSt* <1..211<br>intergenic 212.. >1866
SCoT22-FaSt-R, *Prunus dulcis* cultivar 'Tétényi keményhéjú'							
PQ869798<br>*Pdul_TK_22_497*	497	*P. dulcis genome assembly, chromosome: 3*	OZ022199.1	100	9e-139	98.60	intergenic <1..211<br>*FaSt* 287..>497
SCoT22-FaSt-R, *Prunus dulcis* cultivar 'Tétényi keményhéjú'							
PQ869799<br>*Pdul_TK_22_901*	901	*P. dulcis DEAD-box ATP-dependent RNA helicase 52C-like* (LOC117633584), mRNA	XM_034367242.1	37	3e-171	100.00	Intron <1..>569<br>*FaSt* <1..>189<br>exon <570..>901
SCoT22-FaSt-R, *Prunus dulcis* cultivar 'Tétényi keményhéjú'							
PQ869800<br>*Pdul_TK_22_263*	263	*P. dulcis genome assembly, chromosome: 1*	OZ022197.1	100	6e-133	100.00	*FaSt* <1..>178<br>intergenic 179.. >263
SCoT22-FaSt-R, *Prunus persica* cultivar 'Collins'							
PQ869801<br>*Pper_CO_22_644*	644	*P. dulcis genome assembly, chromosome: 1*	OZ022197.1	100	0.0	95.49	intergenic <1..432<br>*FaSt* 433..>644

**Accession Nu.**	**Size bp**	**BLAST homolog**	**Accession Nu. of BLAST hit**	**Coverage %**	***E* value**	**Identity %**	**Regions (bp)**
SCoT22-FaSt-R, *Prunus domestica* cultivar 'Nemtudom P3'							
PQ869802<br>*Pdom_NT_22_873*	873	*P. dulcis ethylene-responsive transcription factor ERF053-like* (LOC117623168), mRNA	XM_034354046.1	100	0.0	97.48	exon <1..390<br>3' UTR 391..>873<br>*FaSt* <815..>873
SCoT22-FaSt-R, *Prunus domestica* cultivar 'Nemtudom P3'							
PQ869803<br>*Pdom_NT_22_334*	334	*P. dulcis glycosyltransferase BC10-like* (LOC117629155), mRNA	XM_034361660.1	17	7e-14	94.83	intron <1..277<br>278.. >334<br>*FaSt* <1..221
SCoT32-FaSt-R, *Prunus domestica* cultivar 'Toptaste'							
PQ869804<br>*Pdom_TT_32_418*	418	*P. armeniaca genome assembly, chromosome: 1*	OZ046360.2	100	2e-95	97.64	*FaSt* <1..212<br>intergenic 213.. >418
SCoT32-FaSt-R, *Prunus domestica* cultivar 'Haroma'							
PQ869805<br>*Pdom_HR_32_551*	551	*P. armeniaca genome assembly, chromosome: 3*	OZ046362.2	100	3e-178	88.27	*FaSt* <1..204<br>intergenic 205.. >551
SCoT32-FaSt-R, *Prunus domestica* cultivar 'Haroma'							
PQ869806<br>*Pdom_HR_32_1224*	1,224	*P. mume beta-D-glucosyl crocetin beta-1,6-glucosyltransferase-like* (LOC103331154), mRNA	XM_008233760.1	100	0.0	97.14	exon <1..1098<br>3' UTR 1099..>1224<br>*FaSt* 1154..>1224
SCoT33-FaSt-R, *Prunus dulcis* cultivar 'Tuono'							
PQ869807<br>*Pdul_TU_33_590*	590	*P. dulcis genome assembly, chromosome: 5*	OZ022201.1	100	0.0	99.74	Intergenic <1..377<br>*FaSt* 378..>590
SCoT33-FaSt-R, *Prunus dulcis* cultivar 'Tuono'							
PQ869808<br>*Pdul_TU_33_342*	342	*P. dulcis genome assembly, chromosome: 6*	OZ022202.1	50	9e-23	94.81	*FaSt* <1..>75<br>Intergenic 76..>342
SCoT33-FaSt-R, *Prunus domestica* cultivar 'Haroma'							
PQ869809<br>*Pdom_HR_33_1460*	1,460	*P. dulcis leaf rust 10 disease-resistance locus receptor-like protein kinase-like 1.2* (LOC117613970), transcript variant X1, mRNA	XM_034342614.1	44	0.0	96.11	intron <1..338<br>exon 339..977<br>intron 978.. >1460<br>*FaSt* <1243..>1460
